# Supplementary material for: Genome-Wide Identification, Evolutionary Analysis, and Expression Patterns of Cathepsin Superfamily in Black Rockfish (Sebastes schlegelii) following Aeromonas salmonicida Infection
Source: Mar Drugs. 2022 Aug 3;20(8):504. doi: 10.3390/md20080504 (PMC9409823; doi:10.3390/md20080504)
Supplement: Supplementary file 1 [file marinedrugs-20-00504-s001.zip › marinedrugs-1806457-supplementary.pdf]

Supplementary Table. S1. Abbreviations of gene names used in synteny analysis.

Gene abbreviation	Full name
pltp	Phospholipid transfer protein
CTSAa	Cathepsine Aa
ube2c	Ubiquitin-conjugating enzyme E2 C
pcif1	phosphorylated CTD interacting factor 1
tfap2c	Transcription factor AP-2 gamma
eya2	Eyes absent homolog 2
ncoa5	Nuclear receptor coactivator 5
snrpc	U1 small nuclear ribonucleoprotein C
ripor3	RIPOR family member 3
usp47	Ubiquitin carboxyl-terminal hydrolase 47
gli1	Zinc finger protein GLI1
CTSAb	Cathepsine Ab
mmp24	Matrix metalloproteinase-24
tfap2c	Transcription factor AP-2 gamma
fancm	Fanconi anemia group M protein
smyd2a	N-lysine methyltransferase SMYD2-A
CTSBa	Cathepsine Ba
gcfc2	GC-rich sequence DNA-binding factor 2
tfb2m	transcription factor B2, mitochondrial
kctd3	potassium channel tetramerization domain containing 3
esrrg	estrogen related receptor gamma
gata4	GATA binding protein 4
fdft1	farnesyl-diphosphate farnesyltransferase 1
CTSBb	Cathepsine Bb
aida	axin interactor, dorsalization associated
mia3	MIA SH3 domain ER export factor 3
dusp10	dual specificity phosphatase 10
CTSC	Cathepsine C
nox4	NADPH oxidase 4
naalad2	N-acetylated alpha-linked acidic dipeptidase 2
sgpp2	sphingosine-1-phosphate phosphatase 2
farsb	phenylalanyl-tRNA synthetase subunit beta
wdfy1	WD repeat and FYVE domain containing 1
serpine2	serpin family E member 2
slc12a3	solute carrier family 12 member 3
CTSDa	Cathepsine Da
il17c	interleukin 17C

rassf9	Ras association domain-containing protein 9
slc24a1	Sodium/potassium/calcium exchanger 1
hacd3	Very-long-chain (3R)-3-hydroxyacyl-CoA dehydratase 3
kmt5b	Histone-lysine N-methyltransferase KMT5B
sphkap	SPHK1 interactor, AKAP domain containing
nlrc5	NLR family CARD domain containing 5
slc6a15	solute carrier family 6 member 15
CTSDb	Cathepsine Db
mrpl18	mitochondrial ribosomal protein L18
trpt1	tRNA phosphotransferase 1
hnrnph1	heterogeneous nuclear ribonucleoprotein H1
rufy1	RUN and FYVE domain containing 1
spata4	spermatogenesis associated 4
pdgfc	platelet derived growth factor C
tomm5	translocase of outer mitochondrial membrane 5
CTSF	Cathepsine F
kti12	KTI12 chromatin associated homolog
blm	BLM RecQ like helicase
CTSHa	Cathepsine Ha
ankrd34c	ankyrin repeat domain 34C
idh2	Isocitrate dehydrogenase [NAD] subunit 2
pex11a	peroxisomal biogenesis factor 11 alpha
fam174b	family with sequence similarity 174 member B
nr2f2	nuclear receptor subfamily 2 group F member 2
slco3a1	solute carrier organic anion transporter family member 3A1
nudt21	nudix hydrolase 21
cmc2	C-X9-C motif containing 2
CTSHb	Cathepsine Hb
idh2	Isocitrate dehydrogenase [NAD] subunit 2
rrnad1	ribosomal RNA adenine dimethylase domain containing 1
isg20l2	interferon stimulated exonuclease gene 20 like 2
bcan	brevican
hapln2	hyaluronan and proteoglycan link protein 2
scnm1	sodium channel modifier 1
tmod4	tropomodulin 4
hormad1	HORMA domain containing 1
CTSK	Cathepsine K
mccc2	methylcrotonyl-CoA carboxylase subunit 2
dapk1	death associated protein kinase 1
CTSLa	Cathepsine La
kank1	KN motif and ankyrin repeat domains 1
dmrt1	doublesex and mab-3 related transcription factor 1
grsf1	G-rich RNA sequence binding factor 1

smn1	survival of motor neuron 1, telomeric
wdr54	WDR54 – WD repeat domain 54
pacrg	parkin coregulated
qkia	Protein quaking-A
CTSLb	Cathepsine Lb
ythdf2	YTH N6-methyladenosine RNA binding protein 2
asap3	ArfGAP with SH3 domain, ankyrin repeat and PH domain 3
cnksr1	connector enhancer of kinase suppressor of Ras 1
grhl3	grainyhead like transcription factor 3
nipal3	NIPA like domain containing 3
elovl6	ELOVL fatty acid elongase 6
spata4	spermatogenesis associated 4
CTSO	Cathepsine O
aga	aspartylglucosaminidase
neil3	nei like DNA glycosylase 3
sil1	SIL1 nucleotide exchange factor
csnk1a1	casein kinase 1 alpha 1
fbxo38	F-box protein 38
glmp	glycosylated lysosomal membrane protein
msto1	misato mitochondrial distribution and morphology regulator 1
rnf5	ring finger protein 5
nrm	nurim
mterf1	mitochondrial transcription termination factor 1
dus3l	dihydrouridine synthase 3 like
CTSSa	Cathepsine Sa
tert	telomerase reverse transcriptase
bcan	brevican
hapln2	hyaluronan and proteoglycan link protein 2
rhbg	Rh family B glycoprotein
mef2d	myocyte enhancer factor 2D
tmod4	tropomodulin 4
hormad1	HORMA domain containing 1
CTSSb	Cathepsine Sb
ergic3	ERGIC and golgi 3
ahcy	adenosylhomocysteinase
gnas	GNAS complex locus
rab22a	RAB22A, member RAS oncogene family
nelfcd	negative elongation factor complex member C/D
stx16	syntaxin 16
Dlg1	discs large MAGUK scaffold protein 1
cth	cystathionine gamma-lyase
CTSZb	Cathepsine Zb
Cachd1	cache domain containing 1

Eif6	eukaryotic translation initiation factor 6
Stat2	signal transducer and activator of transcription 2
Npepl1	aminopeptidase like 1

Supplementary Table. S2. Abbreviations and gene ID of gene names used in phylogenetic tree Fig. 3

CTSA_Atlantic_Salmon	NP_001133654.1
CTSC_Atlantic_Salmon	XP_014018939.1
CTSD_Atlantic_Salmon	XP_014032104.1
CTSF_Atlantic_Salmon	XP_014068431.1
CTSH_Atlantic_Salmon	XP_013980319.1
CTSB_Atlantic_Salmon	XP_014060893.1
CTSO_Atlantic_Salmon	NP_001134063.1
CTSW_Atlantic_Salmon	NP_001133678.1
CTSZ_Atlantic_Salmon	XP_014022687.1
CTSA_Catfish	XP_017334981.1
CTSC_Catfish	XP_017346971.1
CTSD_Catfish	NP_001244039.1
CTSW_Catfish	XP_017329569.1
CTSH_Catfish	NP_001187181.1
CTSK_Catfish	NP_001187379.1
CTSL_Catfish	NP_001187182.1
CTSO_Catfish	XP_017330673.1
CTSS_Catfish	NP_001187179.1
CTSZ_Catfish	XP_017337363.1
CTSA_Chicken	NP_001026662.2
CTSB_Chicken	NP_990702.3
CTSC_Chicken	NP_001308483.1
CTSD_Chicken	NP_990508.1
CTSE_Chicken	XP_015154556.1
CTSH_Chicken	NP_001305336.2
CTSK_Chicken	NP_990302.3
CTSG_Chicken	XP_423728.3
CTSO_Chicken	NP_001026300.1
CTSS_Chicken	NP_001026516.1
CTSV_Chicken	NP_001161481.1
CTSZ_Chicken	XP_417483.3
CTSA_Fugu	XP_003976913.1
CTSB_Fugu	XP_003971767.2
CTSC_Fugu	XP_011606588.1
CTSD_Fugu	NP_001072052.1
CTSL_Fugu	XP_003970181.1
CTSH_Fugu	XP_003967090.2
CTSK_Fugu	XP_003965855.1
CTSL_Fugu	XP_003975123.1

CTSL-like_Fugu	XP_029688789.1
CTSO_Fugu	XP_011609247.2
CTSS_Fugu	XP_003965856.2
CTSZ_Fugu	XP_003973526.1
CTSA_Mouse	NP_001033581.1
CTSB_Mouse	NP_031824.1
CTSC_Mouse	NP_001298719.1
CTSD_Mouse	NP_034113.1
CTSE_Mouse	NP_031825.2
CTSF_Mouse	NP_063914.1
CTSG_Mouse	NP_031826.1
CTSH_Mouse	NP_001299578.1
CTSJ_Mouse	NP_001343220.1
CTSK_Mouse	NP_031828.2
CTSL_Mouse	NP_034114.1
CTSM_Mouse	NP_001347650.1
CTSO_Mouse	NP_808330.1
CTSQ_Mouse	NP_083912.2
CTSR_Mouse	NP_064680.1
CTSS_Mouse	NP_001254624.2
CTSW_Mouse	NP_034115.2
CTSZ_Mouse	NP_071720.1
CTSA_Human	NP_000299.3
CTSB_Human	NP_001304166.1
CTSC_Human	NP_001107645.1
CTSD_Human	NP_001900.1
CTSE_Human	NP_001304260.1
CTSF_Human	NP_003784.2
CTSG_Human	NP_001902.1
CTSH_Human	NP_001306066.1
CTSK_Human	NP_000387.1
CTSL_Human	NP_001244900.1
CTSO_Human	NP_001325.1
CTSS_Human	NP_001186668.1
CTSV_Human	NP_001188504.1
CTSW_Human	NP_001326.3
CTSZ_Human	NP_001327.2
CTSA_Flounder	XP_019969053.1
CTSC_Flounder	XP_019950816.1
CTSF_Flounder	XP_019938592.1
CTSK_Flounder	XP_019949027.1
CTSL_like_Flounder	XP_019965003.1
CTSL_Flounder	XP_019964915.1

CTSO_Flounder	XP_019936419.1
CTSZ_Flounder	XP_019942181.1
CTSS_like_Flounder	XP_019948991.1
CTSA_Medaka	NP_001287800.1
CTSC_Medaka	XP_004075795.1
CTSD_Medaka	XP_023807545.1
CTSF_Medaka	XP_004073613.1
CTSH_Medaka	XP_023811589.1
CTSK_Medaka	XP_004078392.1
CTSB_Medaka	XP_020555295.2
CTSL_Medaka	XP_004082231.1
CTSO_Medaka	XP_004086527.1
CTSS_Medaka	NP_001098157.1
CTSZ_Medaka	XP_023810942.1
CTSA_Tilapia	NP_001298249.1
CTSC_Tilapia	XP_003441633.1
CTSD_Tilapia	XP_003452633.1
CTSF_Tilapia	XP_003451852.1
CTSAH_Tilapia	XP_003440460.1
CTSK_Tilapia	XP_019220230.1
CTSB_Tilapia	XP_003454569.1
CTSO_Tilapia	XP_003443496.2
CTSZ_Tilapia	XP_003441550.1
CTSK_Rainbow_Trout	XP_036801718.1
CTSBb_Rainbow_Trout	NP_001117776.1
CTSH_Rainbow_Trout	XP_021427100.1
CTSD_Rainbow_Trout	NP_001118183.1
CTSA_Rainbow_Trout	XP_021466360.1
CTSC_Rainbow_Trout	NP_001117966.1
CTSO_Rainbow_Trout	XP_021417079.2
CTSZ_Rainbow_Trout	XP_021465814.2
CTSA_River_Trout	XP_029548870.1
CTSL_like_River_Trout	XP_029586573.1
CTSH_River_Trout	XP_029613466.1
CTSA_Tongue_Sole	XP_008319457.1
CTSC_Tongue_Sole	XP_008308025.1
CTSD_Tongue_Sole	NP_001281160.1
CTSF_Tongue_Sole	XP_008325732.1
CTSH_Tongue_Sole	XP_008309210.1
CTSK_Tongue_Sole	XP_008322033.1
CTSL_Tongue_Sole	XP_024917672.1
CTSB_Tongue_Sole	XP_008311144.1
CTSO_Tongue_Sole	XP_024918766.1

CTSZ_Tongue_Sole	XP_008318331.1
CTSS_Tongue_Sole	XP_008322035.1
CTSA_Turbot	XP_035486752.1
CTSC_Turbot	XP_035479088.1
CTSF_Turbot	XP_035505110.1
CTSH_Turbot	XP_035497764.1
CTSD_Turbot	NP_001072052.1
CTSK_Turbot	XP_003965855.1
CTSL-like_Turbot	XP_035476549.1
CTSL_Turbot	XP_035467019.1
CTSO_Turbot	XP_035506512.1
CTSS_Turbot	XP_035463881.1
CTSZ_Turbot	XP_035488786.1
CTSK_Turbot	XP_035464553.1
CTSA_Zebrafish	NP_956844.1
CTSC_Zebrafish	NP_999887.1
CTSD_Zebrafish	NP_571785.2
CTSF_Zebrafish	NP_001071036.1
CTSH_Zebrafish	NP_997853.1
CTSK_Zebrafish	NP_001017778.1
CTSLb_Zebrafish	NP_571273.2
CTSL-like_Zebrafish	XP_021335957.1
CTSBa_Zebrafish	NP_998501.1
CTSO_Zebrafish	XP_021336916.1
CTSZ_Zebrafish	NP_001006043.1
CTSS_Zebrafish	NP_001019580.2

Supplementary Table. S3. Abbreviations and gene ID of gene names used in PPI

neu1	ENSDARP00000015727
galns	ENSDARP00000124843
ctsd	ENSDARP00000074872
ctsz	ENSDARP00000063250
ctsba	ENSDARP00000071885
glb1	ENSDARP00000106759
TPP2	ENSDARP00000126605
agtrap	ENSDARP00000104107
NAPSA	ENSDARP00000012342
nots	ENSDARP00000069198
hspa5	ENSDARP00000017456
kif1a	ENSDARP00000129632
elna	ENSDARP00000093092
pebp1	ENSDARP00000112865
cd74a	ENSDARP00000002547
TOLL8	LOC100332583
mhc2b	ENSDARP00000072268
mhc2bl	ENSDARP00000088705
zgc:153067	ENSDARP00000090055
si:zfos-2070c2.3	ENSDARP00000097822
mhc2dab	ENSDARP00000100252
mhc2dcb	ENSDARP00000110668
mhc2d8.46a	LOC571282
bida	ENSDARP00000126479
zgc:65811	ENSDARP00000066141
ctsba	ENSDARP00000071885
cst3	ENSDARP00000113296
mhc2a	LOC791723
zgc:153129	ENSDARP00000067593
zgc:66382	ENSDARP00000124424
psap	ENSDARP00000045068
zgc:162351	ENSDARP00000091917
bloc1s6	ENSDARP00000122569
chchd7	ENSDARP00000124392
ifi30	ENSDARP00000073325
prcp	ENSDARP00000077802
ctssb.1	ENSDARP00000099159
scpep1	ENSDARP00000058604
letm1	ENSDARP00000074011

obsl1a	ENSDARP00000122095
obsl1a	ENSDARP00000122095
zfyve28	ENSDARP00000116818
epx	ENSDARP00000010533
TPO	ENSDARP00000046463
lamp1b	LOC563328
igf2r	ENSDARP00000061100
Lamp1	ENSDARP00000070764
lamp2	ENSDARP00000105565
esr1	ENSDARP00000024987
cpvl	ENSDARP00000073441
sftpba	ENSDARP00000122408
cdh22	ENSDARP00000104331
ZXDC	ENSDARP00000126156
cobl	ENSDARP00000108248
IQSEC1	ENSDARP00000043090
il12rb2	ENSDARP00000005204
pdia4	ENSDARP00000018458
mmp13b	ENSDARP00000036104
mmp20a	ENSDARP00000106475
ugt1b5	ENSDARP00000112070
wfikkn1	ENSDARP00000098282
ccr12.2	ENSDARP00000066871
fabp6	ENSDARP00000065447
birc5a	ENSDARP00000101794
birc5b	ENSDARP00000121836
bcl2l1	ENSDARP00000027919
birc2	ENSDARP00000103254
bcl2	ENSDARP00000105564
bcl2b	ENSDARP00000124838
si:busm1-194e12.11	ENSDARP00000072260
si:zfos-2070c2.1	ENSDARP00000072260
si:zfos-2070c2.3	ENSDARP00000097822
txndc5	ENSDARP00000116609
dnase2	ENSDARP00000120160
agt	ENSDARP00000017923
stx17	ENSDARP00000114720
csnk1da	ENSDARP00000003101
bet1	ENSDARP00000010900
csnk1db	ENSDARP00000014900
sly1	ENSDARP00000121390
slmo2	ENSDARP00000020101

Supplementary Table. S4. Abbreviations of gene names used in PPI.

neu1	Neuraminidase 1
galns	Galactosamine (N-acetyl)-6-sulfate sulfatase
ctsd	Cathepsin D
ctsz	Cathepsin Z
ctsba	Cathepsin B, a
glb1	Galactosidase, beta 1
TPP2	Tripeptidyl peptidase II
agtrap	Angiotensin II receptor-associated protein
NAPSA	Napsin A aspartic peptidase
nots	Nothepsin
hspa5	Heat shock protein 5
kif1a	Kinesin family member 1Aa
elna	Elastin a
pebp1	Phosphatidylethanolamine binding protein 1
cd74a	CD74 molecule
TOLL8	Toll-like receptor 8;
mhc2b	Major histocompatibility complex class ii integral membrane beta chain precursor
mhc2bl	H-2 class II histocompatibility antigen, E-S beta chain
zgc:153067	Major histocompatibility complex class II DBB gene
si:zfos-2070c2.3	Rano class ii histocompatibility antigen, a beta chain-like
mhc2dab	Major histocompatibility complex class ii dab precursor
mhc2dcb	Major histocompatibility complex class ii dcb precursor
mhc2d8.46a	H-2 class II histocompatibility antigen
bida	BH3 interacting domain death agonist
zgc:65811	Tetraspanin
ctsba	Cathepsin B, a
cst3	Cystatin C
mhc2a	Novel vertebrate MHC class II alpha chain protein
zgc:153129	tandem duplicate 1
zgc:66382	tandem duplicate 1
psap	Prosaposin
zgc:162351	Major histocompatibility complex class I ZEA
bloc1s6	Biogenesis of lysosome-related organelles complex 1 subunit 6
chchd7	Coiled-coil-helix-coiled-coil-helix domain containing 7
ifi30	Gamma-interferon-inducible lysosomal thiol reductase
prcp	Prolylcarboxypeptidase (angiotensinase C)
ctssb.1	Cathepsin Sb, tandem duplicate 1
scpep1	Serine carboxypeptidase 1

letm1	LETM1 and EF-hand domain-containing protein 1
obsl1a	LETM1 and EF-hand domain-containing protein 1
obsl1a	Obscurin-like protein 1a; Obscurin-like 1a
zfyve28	Lateral signaling target protein 2 homolog
epx	Eosinophil peroxidase
TPO	Thyroid peroxidase
lamp1b	Lysosome-associated membrane glycoprotein 1b precursor
igf2r	Cation-independent mannose-6-phosphate receptor precursor
Lamp1	Lysosomal-associated membrane protein 1
lamp2	Lysosomal-associated membrane protein 2
esr1	Estrogen receptor alpha
cpvl	Probable serine carboxypeptidase cpvl precursor
sftpba	Surfactant protein Ba
cdh22	Cadherin-22-like
ZXDC	ZXD family zinc finger C
cobl	Protein cordon-bleu
IQSEC1	IQ motif and Sec7 domain 1
il12rb2	Interleukin-12 receptor subunit beta-2
pdia4	Protein disulfide-isomerase A4
mmp13b	Matrix metalloproteinase 13b
mmp20a	Matrix metallopeptidase 20a
ugt1b5	UDP glucuronosyltransferase 1 family, polypeptide B5
wfikkn1	Kunitz and NTR domain-containing protein
ccr12.2	Chemokine (c-c motif) receptor 12b, tandem duplicate 2
fabp6	Fatty acid binding protein 6
birc5a	Baculoviral IAP repeat-containing 5a
birc5b	Baculoviral IAP repeat-containing 5B
bcl2l1	Bcl-xL-like protein 1
birc2	Baculoviral iap repeat-containing protein 2/3
bcl2	B-cell leukemia/lymphoma 2
bcl2b	B-cell CLL/lymphoma 2b
si:busm1-194e12.11	Novel protein similar to MHC class II alpha chain
si:zfos-2070c2.1	Uncharacterized protein loc368748 precursor
si:zfos-2070c2.3	Rano class ii histocompatibility antigen, a beta chain-like
txndc5	Thioredoxin domain containing 5
dnase2	Deoxyribonuclease II
agt	Angiotensinogen
stx17	Syntaxin 17
csnk1da	Casein kinase I isoform delta-A
bet1	Blocked early in transport 1
csnk1db	Casein kinase I isoform delta-B

sly1	Suppressor of ypt1
slmo2	Slowmo homolog 2
